# Natural Antioxidants in Foods and Medicinal Plants: Extraction, Assessment and Resources

**DOI:** 10.3390/ijms18010096

**Published:** 2017-01-05

**Authors:** Dong-Ping Xu, Ya Li, Xiao Meng, Tong Zhou, Yue Zhou, Jie Zheng, Jiao-Jiao Zhang, Hua-Bin Li

**Affiliations:** 1 Guangdong Provincial Key Laboratory of Food, Nutrition and Health, School of Public Health, Sun Yat-Sen University, Guangzhou 510080, China; xudp@mail2.sysu.edu.cn (D.-P.X.); liya28@mail2.sysu.edu.cn (Y.L.); mengx7@mail2.sysu.edu.cn (X.M.); zhout43@mail2.sysu.edu.cn (T.Z.); zhouyue3@mail2.sysu.edu.cn (Y.Z.); zhengj37@mail2.sysu.edu.cn (J.Z.); zhangjj46@mail2.sysu.edu.cn (J.-J.Z.); 2 South China Sea Bioresource Exploitation and Utilization Collaborative Innovation Center, Sun Yat-Sen University, Guangzhou 510006, China

**Keywords:** antioxidant, extraction, assessment, resource

## Abstract

Natural antioxidants are widely distributed in food and medicinal plants. These natural antioxidants, especially polyphenols and carotenoids, exhibit a wide range of biological effects, including anti-inflammatory, anti-aging, anti-atherosclerosis and anticancer. The effective extraction and proper assessment of antioxidants from food and medicinal plants are crucial to explore the potential antioxidant sources and promote the application in functional foods, pharmaceuticals and food additives. The present paper provides comprehensive information on the green extraction technologies of natural antioxidants, assessment of antioxidant activity at chemical and cellular based levels and their main resources from food and medicinal plants.

## 1. Introduction

In biological system, reactive oxygen species (ROS) and reactive nitrogen species (RNS), such as superoxide, hydroxyl, and nitric oxide radicals, can damage the DNA and lead to the oxidation of lipid and proteins in cells [1,2,3]. Normally, antioxidant system occurring in human body can scavenge these radicals, which would keep the balance between oxidation and anti-oxidation. Nonetheless, the exposure of cigarette smoking, alcohol, radiation, or environmental toxins induces the production of excessive ROS and RNS, which disrupt the balance between oxidation and anti-oxidation and result in some chronic and degenerative diseases [3,4,5]. The increment of intake of exogenous antioxidants would ameliorate the damage caused by oxidative stress through inhibiting the initiation or propagation of oxidative chain reaction, acting as free radical scavengers, quenchers of singlet oxygen and reducing agents [6].

The exogenous antioxidants are mainly derived from food and medicinal plants, such as fruits, vegetables, cereals, mushrooms, beverages, flowers, spices and traditional medicinal herbs [7,8,9,10,11,12,13,14,15,16,17,18]. Besides, the industries processing agricultural by-products are also potentially important sources of natural antioxidants [19]. These natural antioxidants from plant materials are mainly polyphenols (phenolic acids, flavonoids, anthocyanins, lignans and stilbenes), carotenoids (xanthophylls and carotenes) and vitamins (vitamin E and C) [6,20]. Generally, these natural antioxidants, especially polyphenols and carotenoids, exhibit a wide range of biological effects, such as anti-inflammatory, antibacterial, antiviral, anti-aging, and anticancer [2,20,21,22,23,24,25,26,27,28,29,30,31].

Considering their important health effects, the efficient extraction methods of natural antioxidants, appropriate assessment of antioxidant activity as well as their main resources from food and medicinal plants are drawing great attention in food science and nutrition. To improve the extraction efficiency of antioxidant components from plant materials, several green non-conventional methods have been developed for reducing operational time and usage of organic solvents, such as ultrasound-assisted extraction, microwave-assisted extraction, enzyme-assisted extraction, pressurized liquid extraction, supercritical fluid extraction, high hydrostatic pressure extraction, pulsed electric field extraction and high voltage electrical discharges extraction. Moreover, to further assess the antioxidant capacities of extracts from natural products, especially those frequently consumed by people, different evaluation assays have been developed, e.g., Trolox equivalence antioxidant capacity (TEAC) assay, ferric ion reducing antioxidant power (FRAP) assay, oxygen radical absorbance capacity (ORAC) assay, inhibiting the oxidation of low-density lipoprotein (LDL) assay, cellular antioxidant activity assay, etc. These assays have been used for ranking the antioxidant plants and recommending best antioxidant foods for consumption. This review is aimed at summarizing the extraction methods of natural antioxidants, assessment methods of antioxidant activity and their main resources from food and medicinal plants.

## 2. Extraction Methods of Antioxidants from Foods and Medicinal Plants

**Figure 1:**
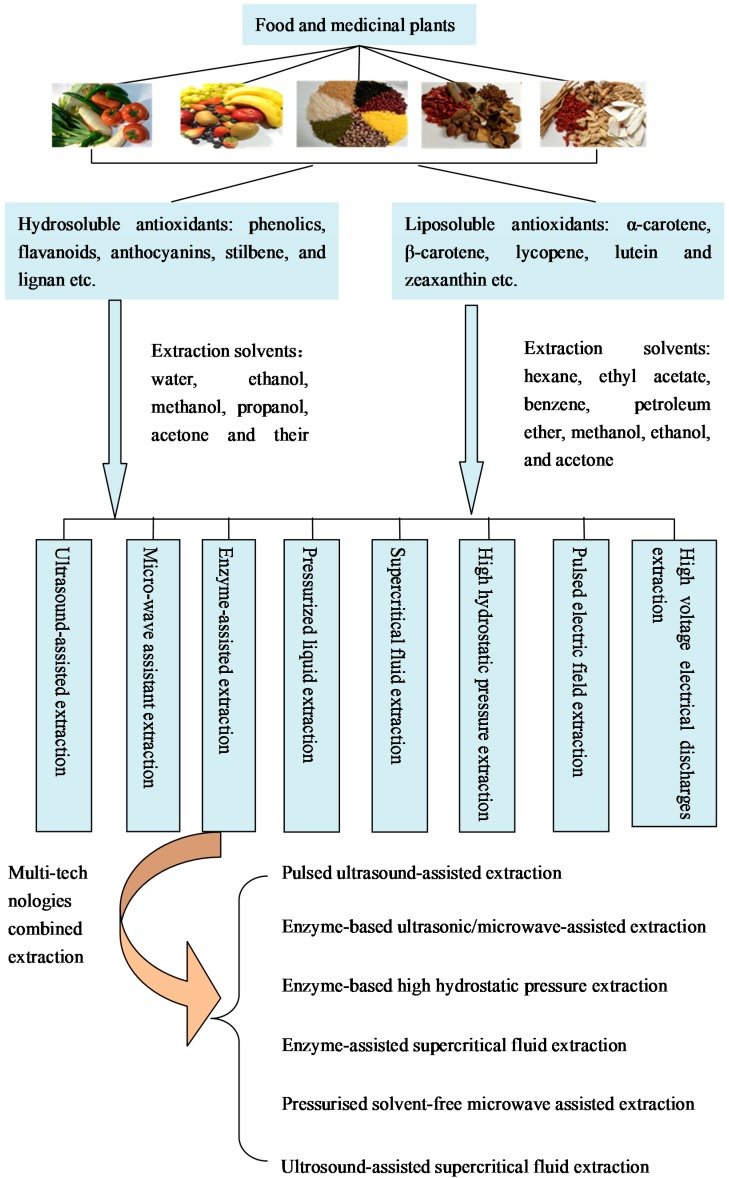
The outline of extraction of antioxidants from foods and medicinal plants.

Extraction is the first and crucial step for studying the natural antioxidants from plants (Figure 1). Many extraction factors play important roles in the extraction efficiency, such as type and concentration of extraction solvent, extraction temperature, extraction time, and extraction pH. Among them, the solvent is one of the most influential factors. Numerous solvents have been used for the extraction of antioxidants from food and medicinal plants. The selection of solvents is based on the chemical nature and polarity of antioxidant compounds to be extracted. Most of the phenolics, flavanoids and anthocyanins are hydrosoluble antioxidants. The polar and medium polar solvents, such as water, ethanol, methanol, propanol, acetone and their aqueous mixtures, are widely used for extraction [32,33,34,35]. Carotenoids are lipid-soluble antioxidants, and common organic solvents, such as the mixtures of hexane with acetone, ethanol, methanol, or mixtures of ethyl acetate with acetone, ethanol, methanol, have been used for extraction [36,37,38].

Various extraction procedures, including conventional extraction methods and non-conventional extraction methods, can be chosen to extract antioxidants from food and medicinal plants. The conventional extraction methods are mainly hot water bath, maceration and Soxhlet extraction, which are very time-consuming and require relatively large amounts of organic solvents with low extraction yields [39,40,41]. Furthermore, the long heating process such as hot water bath and Soxhlet extraction may lead to the degradation of the thermolabile compounds. To obtain antioxidants from plants in an energy-efficient and economically sustainable way, ultrasound, microwave, pressurized liquid, enzyme hydrolysis, supercritical fluids, high hydrostatic pressure, pulsed electric field, and high voltage electrical discharges have been studied as non-conventional methods (Table 1). In this section, the current application and developments of the non-conventional methods are summarized.

### 2.1. Ultrasound-Assisted Extraction (UAE)

Ultrasound assisted extraction (UAE) has been applied widely in the last three decades as an efficient extraction method in the food and pharmaceutical industries [61]. The mechanism is based on the cavitation phenomenon. The spread of ultrasound in liquid systems is via a series of compression and rarefaction waves, which can induce the production of cavitation bubbles within the fluid [62,63]. The size of these bubbles grow over the period of a few cycles until reach a critical point, then these bubbles collapse and release a great quantity of energy, which would generate extreme temperatures (5000 K) and pressures (1000 atmospheres) at room temperature. During the ultrasound assisted extraction of bioactive components from plant materials, the high temperature and pressure would destroy the cell walls, facilitate the release of bioactive compounds from plant cell walls and enhance the mass transport. The heat transfer of UAE is from outside of the plant cell to the inside, which is in the opposite direction of microwave assisted extraction.

Ultrasound frequency, intensity, temperature, and time can directly affect both extraction efficiency and yields. In addition, types of solvent, solvent volume, as well as sample characteristics such as moisture content of the sample and particle size are also the important factors for effective extraction [64]. Ultrasound-assisted extraction of anthocyanins and phenolic compounds in mulberry (*Morus nigra*) pulp was performed by Espada-Bellido et al. [65]. Several extraction variables, including methanol concentration (50%–100%), temperature (10–70 °C), ultrasound amplitude (30%–70%), cycle (0.2–0.7 s), solvent pH (3–7) and solvent–solid ratio (10:1.5–20:1.5) were optimized to obtain the high extraction yields. It was found that extraction temperature and solvent concentration were the most influential parameters for extraction of anthocyanins and phenolic compounds. Besides, different UAE conditions were suitable for different bioactive components. The optimum UAE conditions for anthocyanins are 76% methanol concentration, 12:1.5 solvent–solid ratio, 48 °C extraction temperature, cycle of 0.7 s and 70% ultrasound amplitude at pH 3, while the optimum UAE conditions for phenolic compounds were 61% methanol concentration, 11:1.5 solvent–solid ratio, 64 °C extraction temperature, cycle of 0.7 s and 70% ultrasound amplitude at pH 7. Under the UAE optimal conditions, the maximized extraction yields of total anthocyanins and total phenolic compounds were 149.95 μg/g and 1214.03 μg/g, respectively. Compared with conventional extraction, UAE showed several merits in terms of extraction yield and extraction time. Polyphenols from apple pomace were extracted using conventional extraction and UAE methods [66]. The results showed that ultrasound-assisted extraction increased the catechin equivalents by more than 20% and the release of flavan-3-ols and procyanidins were also enhanced by more than 25% in only 45 min. In another study, Liu et al. [67] used UAE and traditional boiling-water extraction methods to extract antioxidants from Xiao-chai-hu-tang extracts, a Chinese herbal prescription. Compared with traditional boiling-water extraction, the ultrasound-assisted extraction significantly increased the antioxidant activities of Xiao-chai-hu-tang extracts, and significantly enhanced the extraction efficiency of flavonoids and phenolics. In addition, in industrial scale, ultrasound-assisted extraction is regarded as a promising technique to take place of conventional procedure due to the less extraction time, higher extraction yield and lower operating temperature.

To increase extraction yields and reduce the energy consumption, some novel efficient ultrasound-assisted extraction techniques have been developed, such as pulsed ultrasound-assisted technique (PUAE), ultra-turrax based ultrasound-assisted extraction (UT-UAE), and ionic liquid-based ultrasonic-assisted extraction (IL-UAE). PUAE is the ultrasound-assisted extraction under the pulsed mode [68]. The ultrasonic processor is turned on and off intermittently when pulsed extraction is performed, thus causing lower heat generation compared with continuous ultrasound-assisted extraction (CUAE). In this case, PUAE is more suitable for the extraction of thermo-sensitive compounds than CUAE. Moreover, the PUAE technique can lower the risk of technological problems such as erosion of the tip. Pan et al. [69] investigated the effects of CUAE and PUAE as well as conventional solvent extraction on the extraction efficiency of phenolic compounds from pomegranate peel. The observed results have shown that in comparison with conventional solvent extraction, PUAE elevated the extraction yield by 22%, and decreased the extraction time by 87%. Additionally, PUAE had 50% energy saving compared to CUAE. Additionally, the combination of ultra-turrax and UAE has been developed to extract bioactive compounds from plants because ultra-turrax can provide effective mass transfer and facilitate bioactive compounds flow into extraction solvent through producing a narrow and uniform particle size distribution. A UT-UAE method was firstly designed for extracting organic acids from plant materials [70]. The extraction yields of chlorogenic acid, caffeic acid, 3,4-dicaffeoylquinic acid, 3,5-dicaffeoylquinic acid and 4,5-dicaffeoylquinic acid by UT-UAE were 1.13–1.23 folds that of UTE (ultra-turrax extraction) and 1.49–1.65 folds that of UAE. Based on the mass transfer kinetics and particle size distribution experiments, it was observed that UT-UAE could uniform particle distribution and increase extraction speed. Furthermore, the combination of ionic liquids (ILs) with ultrasonic-assisted extraction can produce a synergetic effect. Tan et al. [71] extracted flavonoids from *Apocynum venetum* L. leaves by IL-UAE. Using the imidazolium IL [C_4mim_] [N(CN)_2_] aqueous solution as solvent, a remarkable extraction efficiency of 93.35% were obtained. Compared to water and conventional organic solvents, ILs possess unique characteristics, such as high polarity, negligible volatility, wide electrochemical windows, high ionic conductivity and chemical stability.

### 2.2. Microwave-Assisted Extraction (MAE)

Microwave is an electromagnetic radiation. During MAE, microwave can deliver energy to solvent and plant matrix and the energy can be absorbed by molecules inside plants, particularly the polar molecules. The severe thermal, localized pressures and mechanical stress caused by microwave significantly change the physical properties of the cell walls and finally result in rupture of cell walls and release of target components [72,73]. Since microwave irradiation was applied for the first time in 1986, there have been various studies on MAE in the recovery of the antioxidants from plant materials [37,74,75,76]. MAE is not adaptable for the extraction of the thermally labile antioxidants due to the thermal effect from microwave irradiation, which might result in the reduction of extraction yield. In addition, MAE is only applicable to the extraction solvents that must be able to absorb microwaves.

Factors influencing MAE are the selection of appropriate solvent, the ratio of solvent to material, irradiation temperature, irradiation time, microwave power, and matrix characteristics [77,78]. The selection of solvent should be based on the solubility of analyte of interest and the capacity of solvent to absorb microwaves. The ratio of solvent to material is also an important factor that influences the extraction process. The volume of solvent need to be sufficient to soak the plant material completely in the solvent during the microwave irradiation process, whereas a higher ratio of solvent to material would not exhibit higher extraction efficiencies due to non-uniform distribution and exposure to microwaves. Irradiation temperature is another vital factor that influences the extraction process. The yield of bioactive components can be enhanced with an increase in the irradiation temperature, but there is a risk of destruction of thermolabile components. Irradiation time and microwave power are interactive variables that influence the extraction process. High power can increase the heating effect and decrease the microwave irradiation time, but also result in degradation of thermolabile components. The matrix characteristics such as particle size and the nature of material can influence the extraction of the components to a considerable extent. For example, the finer particle size of the sample can offer the larger surface area and the better penetration [77]. A great number of researches indicated that MAE exerts several advantages in comparison with conventional extraction methods, such as higher extraction yield, less solvent consumption and shorter extraction time [72,79]. Microwave-assisted extraction of polyphenols from *Myrtus communis* L. leaves was performed by Dahmoune et al. [76]. Authors optimized four parameters, ethanol concentration, solvent/solid ratio, irradiation time, and microwave power to obtain the highest extraction of polyphenols. It was concluded that ethanol concentration and liquid-to-solid ratio were the important parameters. In the study, conventional solvent extraction (CSE) took 2 h and consumed 50 mL/g solvent to extract polyphenols from *Myrtus communis* L. leaves, while MAE took only 62 s and consumed 32 mL/g solvent to extract polyphenols [76]. Under the MAE optimal conditions, the extraction yields of total phenolic (162.49 ± 16.95 mg gallic acid equivalent GAE/g dry weight (DW)), total flavonoid (5.02 ± 0.05 mg quercetin equivalents (QE)/g), and condensed tannins (32.65 ± 0.01 mg/g), were higher than those of conventional solvent extraction (128.00 ± 18.07 mg GAE/g DW, 4.15 ± 0.75 mg QE/g, and 17.18 ± 0.01 mg/g, respectively).

Solvent-free microwave extraction (SFME) as a green technique has been developed recently in accordance with the same principle of MAE [80]. The in-situ water in plant cells absorbs the energy and is stimulated to rotate under a microwave treatment. The immediate dramatic change causes a subsequent pressure increase inside plant cells. Then, cell walls are broken down and the target molecules are released. The extraction proceeds under atmospheric pressure. For fresh plant samples, microwave energy is mostly absorbed by water naturally occurred in plant materials, whereas for dried plant samples, these samples should be moistened with water or other solvents, or microwave absorption media prior to extraction, for increasing the absorbance of microwave energy [72,81]. Primarily, SFME was mainly applied in extraction of antioxidant essential oil from aromatic herbs and spices, such as *Rosmarinus officinalis*, *Origanum vulgare* and *AlgerianLaurus nobilis* L. [81,82,83]. Lately, there are several reports regarding the SFME of antioxidants in plant materials, such as flavonoids from onions [84], phenolic antioxidants from *Citrus deliciosa* T. leaves [82], and natural antioxidants from sea buckthorn food by-products [85]. Besides, a protocol of pressurized solvent-free microwave assisted extraction (PSFME) method was developed for extraction of antioxidants from *Hippophae rhamnoides* L. berries [86]. The extracts obtained by PSFME showed the richest phenolic content compared with pressing, maceration and pressurized liquid extraction. Furthermore, MAE coupled with vacuum system has been developed to extract antioxidants from plant materials [72]. The vacuum in the extraction vessel is achieved by vacuum pump. The vacuum microwave assisted extraction can avoid or reduce degradation of thermolabile and oxygen-sensitive phytochemicals from plants compared to conventional open MAE, mainly due to the low O_2_ concentration and moderate or even low temperature in the extraction vessel [72,87]. The extraction of thermo- and oxygen-sensitive components under the vacuum condition usually showed a high extraction yield with low temperature.

### 2.3. Enzyme-Assisted Extraction (EAE)

Enzymes have the properties of high specificity and high efficiency. Enzyme-assisted extraction (EAE) is a potential green extraction method because of the mild extraction conditions and barely any effect on the environment [88]. The enzymes could degrade the compositions and destroy the structural integrity of plant cell wall, which enhance the release of bioactive compounds. Cellulase, pectinase, hemicellulase and β-glucosidase are extensively used in the EAE. These enzymes can be obtained from various materials such as bacteria, fungi, vegetable and fruit extracts, or animal organs [88,89]. EAE techniques have been shown to improve the extraction efficiencies for antioxidants including phenolics, flavonoids, anthocyanins, and carotenoids [47,48,90,91,92].

Types of enzymes, concentration, pH, incubation temperature, incubation time, liquid to solid ratio and particle size are the key factors influencing extraction efficiency and yield [93]. Different enzymes degrade different substrates, and the concentration affects the extraction efficiency. Furthermore, the pH for enzymatic hydrolysis varies from enzyme to enzyme. The pH affects not only enzyme activity but also the dissociation of some bioactive components such as anthocyanin. Besides, liquid to solid ratio, incubation temperature and time are factors that should not be neglected during processing. Therefore, optimum extraction parameters vary from different plant materials and enzymes. Enzyme-assisted extraction of polyphenols from cauliflower (*Brassica oleracea* L. var. botrytis) outer leaves was performed by Nguyen et al. [91]. Different enzyme concentrations (0%–5% enzyme/substrate ratio) for Viscozyme L and Rapidase, incubation temperatures (30 to 50 °C), pH values (3.0 to 6.0) and incubation times (0 to 24 h) were studied. It was concluded that these parameters significantly affected the extraction yield of polyphenols. The optimal extraction processes for the enzyme treatment were: enzyme/substrate ratio of 0.2% for Viscozyme L and 0.5% for Rapidase, temperature 35 °C, and pH 4.0. As compared to the control without enzyme treatment (457 ± 23 mg GAE/100 g DW), the enzymatic treatments had marked impacts on the release of phenolic compounds (581 ± 16 mg GAE/100 g DW using Viscozyme L, 604 ± 9 mg GAE/100 g DW using Rapidase). In another study, an EAE method was applied to the extraction of phenolic compounds from watermelon rind using the mixture of pectinase, protease, α-amylase and β-glycosidase [92]. Four variables, enzyme concentration (0.5%–6.5%), pH (6–9), temperature (25–75 °C) and treatment time (30–90 min), were studied to obtain optimum yield of polyphenols. The results obtained under optimum conditions (2.24% enzyme concentration, 6.58 pH, 51.8 °C, 30 min) indicated that optimized EAE enhanced the release of antioxidant phenolics up to 3 folds as compared to conventional solvent extraction, with level of 173.70 mg GAE/g FW (total phenolics), 279.96 mg TE/g FW (TEAC) and 112.27 mg/mL (DPPH radical scavenging ability (IC_50_)). Nevertheless, the application of EAE exhibits some potential commercial and technical limitations [94]. For example, the price of enzymes is relatively high, which influences the application, and the activity of enzymes varies with the environmental factors such as temperature and nutrient availability, therefore this method needs further improvement for its application in industrial scale.

Recently, EAE coupling with ultrasound, microwave and high hydrostatic pressure extraction technique has been developed for further enhancing extraction yields. For example, Liu et al. [89] studied an enzyme-based ultrasonic/microwave-assisted extraction (EUMAE) method for extracting orcinol glucoside (a phenolic compound) from the rhizomes of *Curculigo orchioides* Gaertn. In this study, enzymatic hydrolysis and ultrasonic/microwave-assisted extraction (UMAE) were combined. Under the optimal extraction conditions, the extraction yield of orcinol glucoside using EUMAE was higher (92.13%) than those of extracts obtained by hot water extraction (65.11%), UAE (76.19%), MAE (73.37%) and UMAE (82.42%). The results indicated that EUMAE showed a higher yield with less energy consumption, and better selectivity. In addition, enzymatic hydrolysis combined with high hydrostatic pressure (HPP) technique was developed for extracting tricin (a flavonoid) from rice hull [95]. The observed results indicated that 0.5% cellulase assisted hydrolysis prior to HPP extraction increased the tricin yield more than twice than that of extract obtained by traditional solvent extraction. Hydrolytic enzymes promote the release of intracellular compounds by rupturing the cell wall compositions and HPP extraction improves the extraction efficiency by increasing solvent permeability.

### 2.4. Pressurized Liquid Extraction (PLE)

PLE is based on the use of solvents at elevated temperature and pressure to extract target components from various matrices [96,97]. By elevating the pressure, the temperature of solvent under liquid state can be above its boiling point at normal temperature, which can enhance mass-transfer rate and promote the solubility of the analytes. The wide ranges of temperature from room temperature to 200 °C and pressure from 35 to 200 bar can be applied in PLE. When the extraction solvent is water, PLE is also called sub-critical water extraction (SWE). When the water is heated to 200–250 °C in SWE, it can be maintained in liquid state, while the dielectric constant (ε) of water is decreased from 80 to 30–25, which is close to the dielectric constant of some organic solvents such as ethanol or methanol. The closed dielectric constants mean the similar polarity of the organic solvent. Although not viable for every application, the use of SWE can be regarded as an effective alternative to organic solvents in some applications. Due to free of organic solvents, SWE is perceived as the “greenest” of the PLEs [97].

The PLE method has been used to increase the extraction yields of various antioxidants from plant materials such as phenolic compounds [98], carotenoids [99], flavonoids [100] and anthocyanins [101]. This technique is a green extraction method due to its reduced solvent usage, less extraction time, less operation steps and light- and oxygen-free environment. The most important parameters in PLE are the extraction solvent, the temperature, the pressure, the static time and the number of cycles [102]. Some parameters such as purge time and flush volume are relatively fixed and high enough to ensure the recovery of components extracted. Several extraction solvents or mixture solvents can be used in PLE for their similarity to that of the target compounds. For example, ethanol was used as the solvent for extraction of polyphenols, while n-hexane was used as the solvent for extraction of β-carotene [51,52]. Temperature and pressure are also important extraction parameters. Higher temperatures decrease the viscosity and the surface tension of liquid solvents, thus allowing better penetration of the matrix particles and allowing the solvent to “wet” the sample matrix more thoroughly, while maximum temperature is limited by the degradation temperature of each bioactive component. Besides, the elevated pressure can force the solvent within the matrix pore to contact the target compounds and extract them. The static extraction time and the number of cycles are also the related extraction parameters. The static extraction time should be long enough to ensure contact between the target components and the solvent, and the number of cycles is the number of times that solvent is put into the extraction cell and kept in contact with the samples. Antioxidants from black bamboo leaves were extracted using PLE with ethanol/water as solvents [51]. Various temperature ranges (40–200 °C), ethanol concentrations (0%–100%), sample sizes (0.425–6 mm), and extraction time (5–25 min) were used to identify the optimal conditions for extracting polyphenols and investigating the antioxidative activity. The results showed that for TP, the best extraction processes were a 0.425 mm size material at 200 °C and 1500 psi with 50% ethanol for 25 min, while, for TF, the best extraction condition was a 4.75 mm size material at 200 °C and 1500 psi with 75% ethanol for 25 min, for DPPH radical scavenging ability, the best extraction processes were a 425 μm size material at 200 °C and 1500 psi with 25% ethanol for 25 min. The optimal PLE method exhibited more than twice the extraction efficiency on the extraction yields (from 240 to 500 mg/1 g dry black bamboo leaves (DL)), TP (from 1510 ± 3.2 to 2682 ± 0.9 mg/100 g DL), and TF (from 182 ± 2.7 to 657 ± 1.7 mg/100 g DL) of the crude extract as compared to the reflux extraction method (~90 °C, 1 L solvent, 60 min), using less extraction solvent (13 mL instead of 1000 mL) in less time (25 min instead of 60 min). The superheated extraction process for black bamboo leaves elevated the antioxidant properties by reducing the solvent viscosity and surface tension as well as disrupting the black bamboo leaves matrix to increase the connection of solvent with phenolics and flavonoids. Furthermore, PLE is an automated extraction process, and the operation is simplified. PLE was compared with manual-liquid extraction for extraction of apple monomeric phenolics [103]. The effects of four independent extraction factors, including extraction solvents (pure methanol and acetone–water (70:30, *v*/*v*)), sample mass (50–550 mg), extraction duration (1–15 min) and number of extraction cycles (1–3) were evaluated on these polyphenol concentrations (HPLC–DAD analysis). It was observed that optimal conditions varied with phenolic compound. For an extraction in favor of chlorogenic acid, hyperoside, quercitrin and ideain, the experimental conditions of two successive extractions with pure methanol from 50 mg freeze-dried apple samples for 15 min using three extraction cycles were recommended. In comparison with manual methods, PLE has a better performance in reduction of the extraction time and the amount of extraction solvent and increase of the extracted phenolic compounds contents. Meanwhile, PLE is suggested as a better extraction method for unstable bioactive compounds affected easily by oxygen and light, such as trans-resveratrol [104]. Pineiro et al. [104] studied the stability of trans-resveratrol from grapes when subjected to the action of the high temperatures employed in PLE. It was observed that the recoveries of resveratrol reached to 99.5% ± 5.7% at the highest assayed temperature (150 °C), which meant that the resveratrol was not degraded using the PLE due to the inert atmosphere employed and the absence of light. However, this technique is not free from limitations. Wianowska et al. [105] found that chlorogenic acid was unstable under PLE conditions. The main caffeoylquinic acid isomer, trans-5-*O*-caffeoylquinic acid, was suffered from isomerization, transesterification, hydrolysis, and reaction with water, even within the short duration of PLE.

### 2.5. Supercritical Fluid Extraction (SFE)

Supercritical fluid extraction (SFE) as a sustainable green technology has been extensively applied since the past decades. Over the critical pressure (pc) and temperature (Tc), the solvent can be transformed into the supercritical state, which shows liquid-like (solvent power, negligible surface tension) and gas-like (elevated diffusivity and low viscosity) properties [106,107,108]. Even though PLE and SFE have in common that they conduct under medium-to-high pressures, SFE operate using solvents at temperatures and pressures above their critical points, whereas PLE is based on the use of liquids at temperatures above their normal boiling points [64]. Compared with normal liquids, supercritical fluids could enhance transport properties, which can diffuse easily through solid materials and therefore obtain faster extraction rates [109]. SFE utilizes the outstanding physicochemical properties of supercritical fluids (SF) to extract target components from various matrices. SFE basically contains two major steps: firstly, the soluble compounds from the plant material are extracted by the supercritical solvent, then these compounds are separated from the supercritical solvent by rapidly reducing the pressure, increasing the temperature, or both [53].

SFE can be affected by numerous factors, such as solvent flow-rate, operating pressure, extraction temperature, amount of modifier, extraction time, whereas origin, particle size, chemical components, as well as the pretreatment conditions also influence the yield and the composition of the extract by SFE [64,110]. These extraction processes can be precisely optimized for maximizing extraction [110]. The selection of best-suited solvent for a certain application is determined by the technical viability, toxicity, cost, and solvation ability. Several solvents have been used as a supercritical solvent, such as propane, ethane, hexane, pentane, butane and carbon dioxide. The amount of solvent used in SFE is greatly smaller than the amount for any extraction process completed under low pressure, which is advocated. CO_2_ is the most frequently used supercritical solvent in SFE since it is nontoxic, available at a reasonable cost, preserves the extracts from atmospheric oxidation and has a moderate critical temperature [109]. Nevertheless, due to its low polarity, CO_2_ cannot completely dissolve high-polarity compounds from plant materials, thus co-solvents (also called modifiers) are generally used to enhance the extraction yield through increasing the solvent polarity of CO_2_. These co-solvents are polar molecules and used in small amounts, which vary from 1% to 15% [53,109]. Ethanol or ethanol aqueous is the mostly used co-solvents.

Studies on the supercritical fluid extraction of caffeic acid derivatives, phenolic compounds, flavonoids, anthocyanin, astaxanthin, carotenoids, lycopodine, terpenoids and other antioxidants from food and medicinal plants have been published [54,107,111,112,113,114,115]. For example, the extraction of anthocyanin and phenolic compounds from jamun fruits [112] was performed by supercritical carbon dioxide extraction. The effects of three independent variables (temperature, pressure and co-solvent flow rate) were estimated on the maximum extraction yield. It was observed that as the pressure increased from 100 to 162 bar, the extraction yields of total monomeric anthocyanin content (TMAC) and total phenolic content (TPC) increased with a mild slope, then the yields slightly decreased when the pressure exceeded 200 bar. As the extraction temperature increased from 40 to 50 °C, the extraction yields of TMAC and TPC increased, but slowly decreased, when the temperature continued to be elevated. Additionally, the co-solvent (99.9% ethanol) flow rate increasing from 1 to 2 g/min caused a higher extraction yield and reached a peak, while the extraction yield had no further significant improvement when co-solvent flow rate exceeded 2 g/min. Finally, pressure of 162 bar, temperature at 50 °C and co-solvent flow rate of 2.0 g/min was selected as optimal values. Under the optimal SFE conditions, 231.28 ± 0.76 mg/100 g for total monomeric anthocyanin content and 1143.051 ± 1.58 mg GAE/100 g for total phenolic content (TPC) were obtained, and seven different anthocyanins as well as eight different bioactive phenolics were identified by HPLC analysis. In a study conducted by Liu et al. [116], the flavonoids from *Calycopteris floribunda* leaves were obtained using supercritical fluid extraction (SFE). The parameters of temperature and pressure were optimized. The results showed that higher total yield of SFE was achieved at lower temperature and higher pressure. The SFE at the CO_2_ flow rate of 1.5 kg/h for approximately 300 min with pressure of 30 MPa at 35 °C was compared with classical organic solvent extraction (CE) with 40% ethanol solution for 8 h at 70 °C. The results showed that the highest global yield (8.01%) was obtained using CE method, whereas the separation efficiency of pachypodol (a potential anticancer compound) from the extracts achieved by SFE was 3.7 times higher than that achieved by CE. The results indicated that SFE could increase the selectivity of some flavonoid components in the extraction process. In the case of carotenoids from persimmon (*Diospyros kaki* L.), the best extraction for xanthophylls (all trans-lutein, all-trans-zeaxanthin and all-trans-beta-cryptoxanthin) were found at 300 bars, 60 °C, and 3 mL/min CO_2_ flow rate, whereas as a non-oxygenated carotenoid, all-trans-beta-carotene was better extracted at 100 bars, 40 °C, and 1 mL/min CO_2_ flow rate [115]. Compared to other extraction methods, SFE have exhibited the higher extraction efficiency and lower extraction time. Talmaciu et al. [117] investigated the effects of different extraction techniques (SFE, UAE and conventional ethanolic extraction) on extraction of polyphenols from spruce bark as a waste from forestry industry. Supercritical fluid extraction was performed at 1200 psi with pure CO_2_ and 70% ethanol as co-solvent for 60 min, UAE was performed using 70% ethanol in an ultrasonic bath at 40 °C for 60 min and the ethanolic extraction was performed using 70% ethanol in a closed oven for 13 days at 40 °C. Results showed that SFE significantly elevated the yield of total phenolic compounds (122.41 mg GAE/g) compared with ethanolic extraction (14.38 mg GAE/g) and UAE (33.48 mg GAE/g). In addition, the analysis of compounds by HPLC showed that SFE extracts contained higher extraction yield of phenolic compounds such as *p*-coumaric acid (260.4073 mg/L), catechin (171.1765 mg/L) and synapic acid (35.0534 mg/L), compared with UAE (99.4731, 112.084, 13.3618 mg/L, respectively). Nonetheless, the high running cost of SFE technique limits the scale-up application. Thus, studies on the economic viability of SFE in large scale can make the SFE process economically sustainable. Solana et al. [118] conducted a large scale of SFE to obtain phenolic and glucosinolate extracts from rocket salad. Results have indicated that the lowest extraction running costs were achieved at 25 MPa and 75 °C, 67% of the total production costs came from the raw materials. The profitability analysis indicated that this method made the process competitive with the price of extracts obtained using organic solvents.

In order to further enhance extraction efficiency and selectivity of targeted compounds, SFE combined with different technologies have been developed [119,120]. Mushtaq et al. [120] developed a comprehensive enzyme-assisted supercritical fluid extraction (EASCFE) for extracting phenolic antioxidants from pomegranate peel. Results showed that the extracts obtained by EASCFE doubled recovery of crude extracts, elevated level of phenolic contents, and enhanced radical scavenging capacity and inhibition of linoleic acid peroxidation in comparison with the control. The appreciable level of vanillic, ferulic, and syringic acids were detected in EASCFE extracts. The pretreatment with enzymes promoted cell wall hydrolysis and supercritical fluid extraction accelerated the mass transfer rate, which both led to a higher extraction yields and selectivity [109,113]. Additionally, the combination of SFE with ultrasound has been also developed. In a study, supercritical CO_2_ assisted by ultrasound was applied to extract antioxidant compounds from blackberry bagasse [119]. It was shown that the application of ultrasound increased its global yield at the pressure of 15 MPa, without affecting the quality of the extracts. Scanning electron microscopy image analyses indicated that ultrasound could rupture the cell walls, facilitating the release of the bioactive compounds. The results confirmed that coupling ultrasound with SFE processes would be a feasible technology to increase extraction efficiency, decrease extraction time and operational cost. Because of the synergistic effects, the combination of several techniques would be promising green processes for the optimal extraction of bioactive components in the future.

### 2.6. Others

High hydrostatic pressure extraction (HHPE) is a novel non-thermal technology to strengthen mass transport [121,122]. During HHPE, the cold isostatic super high hydraulic pressure varies from 100 to 800 MPa or even more up to 1000 MPa. Under high pressure, the great differential pressure between the interior and exterior of cell membranes forms, and this accelerates the solvent penetration into cells through the broken membranes and increases the mass transfer rate, which indicates that the higher the hydrostatic pressure is, the more components can be released and the higher yield of extraction can be obtained [122]. The energy consumption (higher pressure) can enhance the extraction efficiency, while it can also increase the cost. At present, pressures up to 600 MPa have the technical limitation for industrial scale [123]. Main factors influencing the HHPE are pressure, time, temperature, solvent types and liquid/solid ratio. A study was performed by Xi et al. [124] to extract polyphenols from green tea leaves using a high hydrostatic pressure extraction method. Various extraction variables in the HHPE procedure, such as different solvents (acetone, methanol, ethanol and water), pressure (100–600 MPa), holding time (1–10 min), ethanol concentration (0%–100%), and liquid/solid ratio (10:1–25:1 mL/g), were evaluated. The optimal extraction yield of polyphenols (30% ± 1.3%) were obtained under the conditions of 50% of ethanol concentration, 20:1 of liquid/material ratio and 500 MPa of high hydrostatic pressure for 1 min, which were comparable to those of heat reflux extraction for 45 min, ultrasonic extraction for 90 min and extraction at room temperature for 20 h, respectively. These data showed that the HHPE is more effective compared with the conventional extraction ones. Additionally, higher ferulic acid extraction yield from *Angelica sinensis* radix and higher flavonoid and anthocyanins recovery from red grape skin have also been found using HHPE [125,126,127].

Apart from the extraction techniques mentioned above, disintegration of the cell membrane in plant materials by pulsed electric field (PEF) technology also has been suggested as a non-thermal extraction method to enhance the extraction yield of bioactive molecules from plants. Pulsed electric fields technique is based on the application of short duration pulses of high electric field intensity (0.1–50 kV/cm) at room temperature [128]. Electric fields of few to several hundred microseconds are capable of inducing pore formation in the cell membrane, which is called “electroporation”. Based on this, subsequent extraction of bioactive molecules can be carried out [129]. Zderic and Zondervan [129] showed that the increment of temperature did not exceed 10 °C during the extraction of polyphenol from fresh tea leaves using PEF. This slight temperature increment confirmed that pulsed electric field processing provided a non-thermal condition. The improvement of extraction efficiency using PEF technology has been demonstrated in the extraction of antioxidants from food and medicinal plants, such as polyphenols from *Norway spruce* bark [130]; anthocyanin from blueberry processing by-products [131]; quercetin and ellagic acid from *Emblica officinalis* juice [132]; anthocyanin from red cabbage [133]; lutein from *Chlorella vulgaris* [134]; and beta-carotene from carrot pomace [135]. The electric field strength plays an important role in solute extraction. Lopez et al. [136] researched the effects of PEF at 5 and 10 kV/cm on the extraction of phenolic compounds during the fermentation of must of *Tempranillo* grapes. Results showed that the permeabilization of the grape skin using PEF treatment at room temperature increased the anthocyanin and polyphenolic content compared to the control during all the vinification process. The anthocyanins contents improved from 835.29 mg/L to 965.02 mg/L (5 kV/cm) and 1056.06 mg/L (10 kV/cm) with the PEF treatment, while, the total phenolic index increased significantly from 70.65 OD 280 nm to 83.1 OD 280 nm (5 kV/cm) and 87.7 OD 280 nm (10 kV/cm) with the PEF treatment. Additionally, PEF did not influence wine characteristics such as alcohol content, total acidity, reducing sugar concentration, and the ratio between the compositions of the red wine color (tint and yellow, red and blue components).

As another electrical treatment technology, high voltage electrical discharges (HVED) technique has been also developed for biomolecules extraction from various products [137]. Under high-current/high voltage electrical discharge, a plasma channel is formed between two submersed electrodes and the energy can be directly injected into an aqueous solution. It induces physical processes and chemical reactions including the production of shock waves, the emission of high-intensity UV light and the generation of hydroxyl radicals via water photodissociation. Shock waves lead to mechanical rupture of cell membranes, UV light between 200 and 400 nm is mutagenic to cells, and hydroxyl radicals cause oxidative damage of cells, which promote the release of bioactive compounds from inside cells [137]. The method has been applied to vegetative raw material, such as vine shoot, green tea, linseed, sesame seed and root of *Datura Innoxia* [138,139,140]. The high voltage electrical discharges assisted extraction is a green extraction technique as it can enhance the rate of extracted biocompounds at reduced time, low temperature and energy input [137]. Boussetta et al. [138] investigated the effects of high voltage electrical discharges (HVED) on the aqueous extraction of polyphenols from grape pomace (*Vitis vinifera* L.) at mild temperature (20–60 °C). The results showed that the yield of extracted solutes (70%) from fresh grape pomace with HVED extraction after 40 min was more than twice the yield (about 30%) obtained after 240 min without HVED. HVED after 1 h also increased the yield of polyphenols (*Y*_polyphenols_ = 0.44% ± 0.07%) compared to that (*Y*_polyphenols_ = 0.26% ± 0.06%) of extraction without HVED after 4 h. Nevertheless, this method may be accompanied by some negative effects. Boussetta and Vorobiev [130] reported that the use of HVED can produce very small particles, which can render the subsequent solid to liquid separation step more difficult.

How to choose a suitable extraction process for natural antioxidant(s): The selection of a suitable extraction process for natural antioxidant(s) is the result of a comprehensive consideration regarding extraction efficiency, economic feasibility and environment aspects of non-conventional extraction techniques. Comparison of non-conventional extraction techniques is displayed in Table 2. Extraction efficiency of non-conventional extraction techniques is clearly advantageous compared with the conventional extraction methods in case of time, energy, and extraction yield. Nevertheless, the extraction efficiencies among non-conventional extraction techniques are different (Table 2). In a study, the comparison of extraction efficiencies between UAE and EAE was carried out by Le et al. [141]. It was observed that the levels of vitamin C, phenolics and antioxidant activity evaluated by DPPH and 2,2′-azinobis-(3-ethylbenzothiazoline-6-sulphonic acid) (ABTS) methods in UAE were, respectively, 4.6%, 3.5%, 4.6% and 3.3% higher than those in EAE. Obviously, UAE is more effective for the extraction of antioxidant compounds from acerola (*Malpighia emarginata* DC.) fruit. In another study, the comparison of extraction efficiencies between UAE and MAE was conducted [74]. The results showed that the optimized MAE gave higher extraction yields compared with UAE in case of total phenolic, total flavonoids and tannins content from the leaves of *Pistacia lentiscus* L. Furthermore, Rodriguez-Perez et al. [142] emphasized that in the extraction of kaempferol, quercetin, and their glucosides derivatives, MAE seemed to be more efficient compared to PLE, whereas PLE was a better choice for the extraction of phenolic compounds with a higher number of hydroxyl-type substituents such as kaempferol diglycoside and its acetyl derivatives as well as those that are sensitive to high temperatures (glucosinolates or amino acids). Moreover, Rosello-Soto et al. [61] indicated that HVEDE was more effective than UAE and PEFE in case of polyphenol extraction (255 mg GAE/L for HVED compared with 140 and 146 mg GAE/L for US and PEF, respectively).

Apart from possessing high extraction efficiency, the extraction methods must show their economic feasibility. Factors affecting the investment cost include direct manufacturing costs (e.g., instrument, raw materials and operational labor), fixed costs (e.g., depreciation and insurance), as well as general expenses (e.g., administration costs and development) [64]. The overall investment costs for the extraction of antioxidants using non-conventional techniques can be high or low (Table 2). UAE and MAE is recommended as the most feasible and economically profitable extraction method in the large scale due to not high investment cost as well as competitive energy efficiency [62]. Although the potential of PLE, SFE, HHPE, PEFE and HVEDE for energy efficient can be clearly observed, the high capital, maintenance and service costs make their application keep in lab- or pilot-scale [123].

Based on the green chemistry and sustainable development concepts, many efforts have been made to provide not only highly efficient, but also eco-friendly methods for the extraction of antioxidants from food and medicinal plants [64]. These non-conventional extraction technologies are conducted with less or no usage of harmful organic solvents. Among non-conventional extraction technologies mentioned above, supercritical fluid extraction, especially supercritical CO_2_ extraction and sub-critical water extraction are recommended. The amount of solvent used in SFE is greatly smaller than that of any extraction process completed under low pressure. CO_2_ is the most frequently used supercritical solvent in SFE since it is nontoxic and available at a reasonable cost [109]. Although not viable for every application, the use of SWE can be regarded as an effective alternative to organic solvents in some applications. Due to free of organic solvents, SWE is perceived as the ”greenest” of the PLEs [97].

How to design extraction process of natural antioxidant(s): Different types of antioxidants (phenolic acids, flavonoids, anthocyanins and carotenoids) can be extracted using non-conventional extraction techniques through adjusting extraction conditions according to the characteristics of components and matrices. Numerous factors have impacts on an extraction process, such as solvent choice, temperature, particle size of sample, pressure, extraction time. Optimization of the process conditions is important for achieving high extraction efficiency and making the final product suitable for different applications. In the preliminary/single factor study, the influence of factors on extraction efficiency/bioactivity needs to be investigated independently. The variables that affect the extraction efficiency/bioactivity significantly are selected for subsequent optimization design. To optimize the significant factors and obtain optimum results with minimum experimental trials, screening experimental designs and optimization experimental designs are used [110]. Screening designs can be used to analyze the most important factors and their interactions from all potential factors. Two-level full factorial, two-level factorial and Plackett–Burman design are frequently used for screening purposes. Optimization designs are used to confirm the optimal conditions of the experiment. Central composite design (CCD), Box–Behnken design (BBD), Taguchi design and Doehlert design are frequently used for optimization purposes [110,143].

## 3. Assessment Methods of Antioxidant Capacity

Assessment of antioxidant capacity of natural products has been regarded as a basis for ranking the antioxidant plants and recommending best antioxidant foods for consumption. The evaluation of antioxidant activity of food and medicinal plants can be performed using chemical-based assays and cellular-based assays.

### 3.1. Chemical-Based Assays

Numerous chemical-based assays have been developed to evaluate the activity of antioxidants in foods and medicinal plants. These assays can roughly be classified into two types according to the mechanism: single electron transfer (SET) and hydrogen atom transfer (HAT) [148,149,150]. SET-based methods measure the ability of an antioxidant to transfer one electron to reduce target charged compounds, such as radicals, and metal ions. Among these SET-based assays, some assays are based on the ability to scavenge the stable free radicals, such as Trolox equivalence antioxidant capacity (TEAC), DPPH assay and Folin–Ciocalteu regent assay, and some assays are based on the ability to reduce metal ions, such as ferric ion reducing antioxidant power (FRAP), and cupric reducing antioxidant capacity (CUPRAC). Meanwhile, HAT-based assays detect the ability of an antioxidant to quench free radicals by hydrogen donation, which are more relevant to the radical chain-breaking antioxidant capacity. HAT-based assays include oxygen radical absorbance capacity (ORAC), total radical trapping antioxidant parameter (TRAP), and inhibiting the oxidation of low-density lipoprotein (LDL).

#### 3.1.1. Scavenging Free Radicals Assays

The Trolox equivalent antioxidant capacity (TEAC) assay is widely applied to evaluate the antioxidant ability to scavenge the ABTS radical [148,149,151,152]. According to the type of oxidation agent, there are two versions of this assay [153]. TEAC1: metmyoglobin-H_2_O_2_ oxidize ABTS to generate its colored ABTS•^+^ form; then, subsequent addition of antioxidants results in loss of the green color. TEAC2: potassium persulfate oxidize ABTS to generate its colored ABTS•^+^ form; then, subsequent addition of antioxidants results in loss of the green color. The version of TEAC1 is inaccurate because antioxidants also can react with the original HO• radical and the metmyoglobin except for the ABTS•^+^, which could cause an overestimation of antioxidant capacity. Therefore, the version of TEAC2 is more preferable. ABTS•^+^ has a UV-vis absorption maximum at 734 nm. The decrease of absorbance can be monitored spectrophotometrically. The difference of the absorbance tested is plotted versus the antioxidants concentrations. The antioxidant capacity was expressed as Trolox equivalents. Because ABTS•^+^ could react rapidly with antioxidants, the assay possesses the merits of rapidity and simplicity. Additionally, ABTS•^+^ is not influenced by ionic strength and is solvable in both organic and aqueous solvents, so it can be applied in multiple media to detect both hydrophilic and lipophilic antioxidant activities [154]. However, for slow reactions, the TEAC values tested is inaccurate when the duration of reaction is beyond 6 min [149].

As one of the few stable organic nitrogen radicals, the 2,2-diphenyl-1-picrylhydrazyl (DPPH) radical is used to analyze the antioxidant activity [151,155]. The DPPH• posses a deep purple color and has a UV-vis absorption maximum at 515 nm [148,149]. The test compounds (antioxidants) reduce DPPH radical to DPPH-H and the solution color fades. The reducing ability can be assessed by measuring the decrease of its absorbance. In the end, the results are shown by EC_50_ and TEC_50_, that is, the necessary amount of antioxidant to decrease the initial DPPH concentration by 50% and the time taken to reach the steady state to EC_50_ concentration [155]. DPPH assay is widely used in antioxidant capacity screening of fruit and vegetable juices or extracts, for it is easy, rapid and requires only a UV-vis spectrophotometer to test. Compared with ABTS assay, the DPPH radical is commercially available and does not have to be generated before assay such as ABTS•^+^. However, the application of DPPH assay is limited by its disadvantage. The linear reaction range of DPPH assay is narrow, only 2–3-fold. Moreover, for steric inaccessibility, antioxidants that possess strong antioxidant activities in lipid peroxidation system may react slowly or may even be inert to DPPH [148,149].

#### 3.1.2. Reducing the Metal Ions Assays (FRAP and CUPRAC Assays)

The ferric-reducing antioxidant power (FRAP) assay measures directly the reducing capacity of antioxidants. In ferric-reducing antioxidant reactive system, the antioxidants can reduce a ferric tripyridyltriazine complex (Fe^3+^-TPTZ) to the ferrous complex (Fe^2+^-TPTZ) under pH 3.6 condition with a blank sample in parallel. The ferrous complex (Fe^2+^-TPTZ) is blue ferrous form and has a UV-vis absorption maximum at 593 nm. The ability of antioxidants in samples (FRAP value) is positive related to the increase in absorbance [151,156,157,158]. In general, this assay is suitable for some antioxidants that complete the reaction rapidly within 4 to 6 min, such as ascorbic acid and uric acid [159]. However, it has been demonstrated that the absorption of several dietary polyphenols in water and methanol slowly increased even after several hours, such as tannic acid, and caffeic acid [157]. In addition, FRAP cannot detect compounds that act by radical quenching (H transfer), particularly thiols and proteins. This results in a serious underestimation in serum sample [149].

The cupric reducing antioxidant capacity (CUPRAC) assay is similar to the FRAP method. CUPRAC method is conducted by mixing Cu(II)-neocuproine (Nc) chelate with antioxidant solution. The absorbance of the color Cu(I)-chelate as a result of redox reaction is measured at 450 nm after 30 min [160]. The application on this assay is less extensive than FRAP. However, this assay exhibits several merits in some ways. For example, the reagent in this assay is useful at pH 7, which is at physiological pH (as opposed to the Folin and FRAP assays, which work at pH 10 and pH 3.6, respectively). This method could be applied for the determination of both hydrophilic and lipophilic antioxidants because the Cu(II)-Nc is soluble in both aqueous and organic environments (unlike Folin and DPPH) [160,161]. In addition, CUPRAC assay can measure the reducing power of thiol-type antioxidants, such as glutathione and nonprotein thiols [150,162].

#### 3.1.3. Folin–Ciocalteu Reagent (FCR) Assay

Folin–Ciocalteu reagent (FCR) assay is a widespread method for quantitative determination of phenolic compounds. The mechanism of Folin–Ciocalteu method is electron transfer (ET) [134,135]. It involves reducibility of phenols in alkaline solution (pH = 10), which is capable of turning yellow molybdotungsto-phosphoric heteropolyanion reagent into the blue resultant molybdotungsto-phosphate [148,163,164]. These blue pigments have a maximum absorption in the 700–760 nm range. The maximum absorption depends on the qualitative and/or quantitative composition of phenolic mixtures. The total phenols assay by FCR is simple, convenient, and has produced a large body of comparable data. Thus, it has become a routine assay in studying phenolic antioxidants from fruits, vegetables and medicine plants [163,165,166,167,168]. A large number of publications found excellent linear correlations between the “total phenolic profiles” by FCR and “the antioxidant activity” by other ET-based antioxidant capacity assay (e.g., FRAP, TEAC, etc.) [148,169,170].

#### 3.1.4. Oxygen Radical Absorbance Capacity (ORAC) Assay

Generally, these assays estimate the capacity of antioxidants to protect a target molecule exposed to a free radical source. The oxygen radical absorbance capacity (ORAC) assay has been applied widely in the field of antioxidant and oxidative stress via H atom transfer [148,149,171,172]. In the basic assay, the peroxyl radical mixes with a fluorescent probe (FL; 3′,6′-dihydroxyspiro[isobenzofuran-1[3H],9′[9H]-xanthen]-3-one), then form a nonfluorescent product, which can be quantitated easily by fluorescence [149,171,173]. When an antioxidant is added into the mixture, peroxyl radical induced oxidation is inhibited and the decay of FL is prevented. Antioxidant capacity is reflected by determining the decreased rate and amount of product formed over time. Using AUC (area under curve) to reflect the antioxidant capacity is favorable because it applies to an antioxidant that has a lag phase or one that do not [171]. It is useful for a broad range of sample types, including raw fruit and vegetable extracts, plasma, and pure phytochemicals. Furthermore, the high-throughput assay is able to test several hundred samples daily by just using one plate-reader coupled with a multichannel automatic liquid handling system [148].

#### 3.1.5. Total Radical Trapping Antioxidant Potential (TRAP) Assay

The total radical trapping antioxidant potential (TRAP) assay measures the ability of antioxidants to suppress the oxidation progress of 2,2′-azobis-2-methyl-propanimidamide, dihydrochloride (AAPH) or 2,2′-azobis(2-amidinopropane) dihydrochloride (ABAP) [148,174]. The variation in the reaction progress is monitored fluorometrically (*λ*_ex_ = 495 nm and *λ*_em_ = 575 nm). The fluorescence decay rate in the reaction slows after the addition of antioxidants compared with the rate before the antioxidants addition. The quantification is based on the lag-phase duration compared with the lag phase of Trolox [149,162]. The application of the lag phase is based on the assumption that the antioxidants show a lag phase and the length of the lag phase is positively correlated to antioxidant capacity. However, not every antioxidant component possesses an obvious lag phase and the potential of antioxidants that play a role after the lag phase is totally ignored.

#### 3.1.6. Inhibiting the Oxidation of Low-Density Lipoprotein (LDL) Assay

Inhibition of induced lipid autoxidation has been developed as a measure of antioxidant capacity in a more physiologically relevant system [148,149,150]. Usually, the reaction solution contains free radical initiator (Cu(II) or 2,2′-azobis(2-amidinopropane) dihydrochloride (AAPH)), substrate (linoleic acid or LDL), and antioxidants. The autoxidation of linoleic acid or LDL is induced by Cu(II) or AAPH. The peroxidation of the lipid components is monitored at 234 nm by UV spectrometer for conjugated dienes. In the presence of a radical initiator, the reaction starts and the absorbance at 234 nm increases as the evidence of the accumulation of conjugated diene oxides. After the addition of antioxidants, the reaction rate slows down until the antioxidant is exhausted. In the period, the lag time is measured and used to evaluate antioxidant capacity.

Compared with other in vitro assays, the major advantage of this method is the use of a biological relevant substrate, which makes the results relevant to oxidative reactions in vivo [149]. Because LDL is isolated from blood samples, one of the major flaws of this method is the variability of the LDL samples, which can vary with different donors. Thus, this method is hard to be developed as a consistent, reproducible, high throughput antioxidant evaluation assay [148,149,150]. On the contrary, using linoleic acid or its methyl ester as an oxidation substrate would make the results more reproducible than using LDL. However, linoleic acid would form micelles in the presence of water, and the reaction progress in micelles cannot be monitored directly by UV absorbance, thus the accuracy of the method can be affected.

### 3.2. Cellular-Based Assays

The antioxidant capacity evaluated by chemical assays cannot completely reflect the behavior of the sample in vivo. It is necessary to estimate the effectiveness of antioxidants in more biologically relevant conditions. Animal models and human studies are more suitable for evaluation but more expensive and time-consuming [150]. As intermediate testing methods, cellular antioxidant activity (CAA) assay has been developed for evaluating the antioxidant capacities [175,176]. Dichlorofluorecin (DCFH) method is a commonly used CAA assay, which tests the capacity of antioxidants to prevent the oxidation of DCFH. In general, DCFH trapped within cells is easily oxidized to fluorescent dichlorofluorescein (DCF) by ABAP-generated peroxyl radicals in human hepatocarcinoma HepG2 cells. DCF could be monitored by fluorescence (λ_exc_ = 485 nm, λ_em_ = 538 nm). The decrease in cellular fluorescence is proportional to the antioxidant capacity of bioactive components. Except for HepG2 cells, several cell types have been applied for the CAA assay, such as human red blood cell, human endothelial EA.hy926, human colon cancer Caco-2 cell, human macrophage U937 cell and mouse macrophage RAW264.7 cell [150,176,177]. Besides, a CAA assay based on microfluidic cell chip with arrayed microchannels has been developed to assess plant antioxidants [178]. The microfluidic chip contains 288 round cell culture micro chambers and 48 independent parallel array channels. In this method, eight groups of different samples with six different concentrations could be tested simultaneously with multimode reader.

The assessment of antioxidant activity at cellular level is not limited to the test of ability of ROS/RNS scavenging but also includes tests of expression of antioxidant enzymes, inhibition of pro-oxidant enzymes, and activation vs. repression of redox transcription factors [150]. The antioxidant activities of the extracts prepared from five brown seaweeds was assessed in Caco-2 cells. Glutathione (GSH) content and antioxidant enzyme activity (catalase (CAT) and superoxide dismutase (SOD)) were evaluated. These cellular assays indicated that *Pelvetia canaliculata* could exert the antioxidant capacity mainly by preventing H_2_O_2_-mediated SOD depletion in Caco-2 cells [179]. Besides, antioxidant enzyme activities of glutathione peroxidase (GPx) and glutathione reductase (GR) were measured in three Argentinean red wines. Some protective effects of wine were observed in cells exposed to H_2_O_2_, which was attributed to the increased activity of antioxidant enzymes GPx and GR [180]. In addition, suppression of NF-κB activation as an anti-oxidant response has been observed in cultured cells with the treatment of phenols (e.g., curcumin) or food extracts (e.g., blueberries) [150,181,182]. In a study, it was observed that the activation of NF-κB and activator protein-1, as well as IL-8 release were suppressed in curcumin-treated alveolar epithelial cells. Additionally, in comparison with untreated cells, the levels of GSH and glutamylcysteine ligase catalytic subunit mRNA expression were increased [181].

## 4. Main Resources of Natural Antioxidants

Among various chemical based assays, the TEAC assay evaluates the ability to scavenge the free radical, the FRAP assay measures directly the reducing capacity of antioxidants, the total phenols assay by FCR assesses the phenolic contents from tested samples. To comprehensively study different aspects of antioxidants, the combination of TEAC, FRAP and FCR methods is frequently used to evaluate the antioxidant activity. As shown in Table 3, the antioxidant activities of many food and medicinal plants have been widely estimated, e.g., fruits, vegetables, cereal grains, edible and wild flowers, macro-fungi, medicinal plants, spices, etc., and the varieties showing strong antioxidant activities were displayed in Table 3 based on a combinative consideration of the results obtained by FRAP, TEAC and FCR assays. Overall, these results showed that different categories exhibited diverse antioxidant capacities and the variation was very large. The FRAP, TEAC and FCR values of 62 fruits varied from 0.11 ± 0.01 to 72.11 ± 2.19 μmol Fe(II)/g, 0.84 ± 0.03 to 80.68 ± 2.11 μmol Trolox/g, and 11.88 ± 0.11 to 585.52 ± 18.59 mg GAE/100 g, respectively [11]. The FRAP, TEAC and FCR values of 56 vegetables varied from 2.69 to 60.9 μmol Fe(II)/g, 6.93 to 33.63 μmol Trolox/g, and 4.99 to 23.27 mg GAE/g, respectively [14]. The FRAP, TEAC and FCR values of 24 cereal grains varied from 5.23 ± 0.23 to 126.19 ± 2.91 μmol Fe(II)/g, 0.62 ± 0.14 to 30.03 ± 1.10 μmol trolox/g, and 1.35 ± 0.15 to 9.47 ± 0.48 mg GAE/g, respectively [12]. The FRAP, TEAC and FCR values of 223 medicinal plants varied from 0.14 to 1844.85 μmol Fe(II)/g, 0.99 to 1544.38 μmol Trolox/g, and 0.19 to 101.33 mg GAE/g, respectively [15]. Obviously, among these varieties showing strong antioxidant activities, the antioxidant activities and total phenolic contents of medicinal plants were significantly higher than those of fruits, vegetables and cereals.

Additionally, the antioxidant activities of food and medicinal plants have also been evaluated by cellular antioxidant activity assays based on different cell types, and the varieties showing strong antioxidant activities were displayed in Table 3. The cellular antioxidant activities of 27 vegetables ranged from not detected (tomato) to 41.9 ± 6.2 μmol of QE/100 g (beet) [183]. The cellular antioxidant activities of 25 fruits ranged from 3.15 ± 0.21 (banana) to 292 ± 11 μmol of QE/100 g (wild blueberry) [184]. In both studies, these results showed that CAA values were significantly associated with total phenolic content. Surarit et al. [185] reported that the ethanolic bran extracts of 11 Thai pigmented (red and purple) and two non-pigmented rice varieties exerted the cellular antioxidant activities based on HL-60 cells in the following order: red > purple > non-pigmented rice, which showed the same order of phenolic and flavonoid contents in these rice extracts.

### 4.1. Natural Sources of Polyphenols

Polyphenols are abundant in food and medicinal plants, including phenolic acids, flavonoids, lignans, and stilbenes [20].

Phenolic acids comprise of derivatives of cinnamic acid (e.g., p-coumaric, caffeic, and ferulic) and derivatives of benzoic acid (e.g., gallic acid and hydroxybenzoic acids). Compared with the hydroxybenzoic acids, the hydroxycinnamic acids are more abundant in edible plants [20]. Fruits such as blueberries, kiwis, plums, cherries, apples are found to be rich in the hydroxycinnamic acids (0.5–2 g hydroxycinnamic acids/kg fresh wt). Caffeic acid is the most abundant phenolic acid and account for 75%–100% of the total hydroxycinnamic acid content in many fruits, whereas, ferulic acid is the most abundant phenolic acid in cereal grains and account for about 90% of total polyphenol content of wheat grain. The hydroxybenzoic acid content in edible plants is usually very low, except for certain red fruits, black radish, and onions. Due to the low content, they are not considered to be of great nutritional interest.

Flavonoids are abundant in most edible fruits and vegetables. Its subclasses include flavonols, flavanones, catechins, flavones, anthocyanidins and isoflavonoids. The concentration and type of flavonoids vary in different dietary sources [186]. Quercetin is usually the most abundant flavonol in edible plants. The richest dietary source of quercetin is onion. Tea and wine have relatively low amounts of quercetin. Other flavonols include myricetin (berries), isorhamnetin (onion), and kaempferol (broccoli). Flavanones almost only exist in citrus fruits. Hesperidin and narirutin are the main flavonoids of oranges and mandarins, whereas, naringin and narirutin are the main flavonoids of grapefruit. Catechins usually exist in the form of aglycones or esterified with gallic acid. The richest dietary sources of catechins are tea and red wine. In addition, the major flavones are apigenin and luteolin. The major dietary sources are red pepper and celery. Anthocyanins, such as pelargonidin, cyanidin, and delphinidin, contribute to the red, blue, or violet color of edible plants, such as plums, eggplant, and many berries. The isoflavonoids, such as isoflavones genistein and daidzein, mainly exist in legumes. The richest dietary source are soybean and soy products [186,187].

The richest dietary source of lignans is linseed containing secoisolariciresinol (up to 3.7 g/kg dry wt) and low quantities of matairesinol [20]. Other algae, leguminous plants (lentils), cereals (triticale and wheat), fruit (pears, prunes) and certain vegetables (garlic, asparagus, carrots) also have traces of these same lignans. Resveratrol is a stilbene, which has been extensively studied for its multiple bioactivities. Red wine is rich in resveratrol (0.3–7 mg aglycones/L and 15 mg glycosides/L).

### 4.2. Natural Sources of Carotenoids

Carotenoids are natural pigments, including β-carotene, lycopene, lutein, and zeaxanthin [188]. All colorful edible plants, especially dark green and yellow-orange leafy, are the good sources of carotenoids. Due to the lipid solubility of carotenoids, the absorption mainly depends on their preparation with oils or fats. Among the carotenoids, the β-carotene commonly occurs in edible plants that possesses the highest activity of provitamin A, such as acerola, mango, pumpkin, carrot, nuts, and oil palm. Lycopene is a kind of red pigment. It almost exists only in vegetables and algae tissues. Tomato products such as juices, soups, sauces, and ketchup, as well as their processing waste and peel are important sources of lycopene [189]. The lycopene (79%–91%) presented in tomatoes is mostly in the form of the trans isomer. Lutein and zeaxanthin are the most common xanthophylls in green and dark leafy vegetables such as broccoli, spinach, peas and lettuce [190]. Zeaxanthin also found in the red marine microalga *P. cruentum* (making up 97.4% of the total carotenoids) (Mezzomo et al., 2016; Raposo et al., 2015) [188,191].

## 5. Conclusions

Antioxidants derived from food and medicinal plants have been increasingly investigated for their various nutritional function and health benefits. In this review, the extraction methods of natural antioxidants, assessment methods of antioxidant activity as well as their main resources from food and medicinal plants are summarized. The non-conventional techniques described have potential to replace or enhance existing extraction techniques due to the less extraction time, energy consumption, and usage of harmful organic solvents, as well as higher extraction yields to recover antioxidant compounds from food and medicinal plants. Nevertheless, most of them are limited for industrial applications due to the high equipment costs and complicated installation procedures. Thus, establishing the balance between energetic and cost will be a key research in the future. To take advantage of the different extraction methods and to limit their drawbacks, the combinative application of multiple extraction technologies and the automated potential of these non-conventional extraction technologies would be the development tendency in future. For assessing the antioxidant activity of plant materials, several assays are suggested, such as the determination of total polyphenolic content by FCR, scavenging free radical ability by TEAC, metal-reducing activity by FRAP, and a kind of cellular-based assay. Furthermore, in order to make the comparison of different samples and studies possible, standardizing the operating conditions of the same analysis method and the expression of results are recommended.

## Figures and Tables

**Table 1:** The application of non-conventional techniques in the extraction of antioxidants from some foods and medicinal plants.

Source	Compounds Extracted	Extraction Parameters		Extraction Improvement	Reference
		Non-Conventional Method	Conventional Methods		
**ultrasound-assisted extraction (UAE)**					
blueberry wine pomace	anthocyanins and phenolics	solvents: 70% ethanol and 0.01% hydrochloric acid; conditions: 400 w, 61.03 °C, 23.67 min	70% ethanol and 0.01% hydrochloric acid; 61 °C, 35 min without ultrasound treatment	increased total anthocyanins from 1.72 to 4.27 mg C_3_G/g (2.5-fold) and total phenolics from 5.08 to 16.41 mg gallic acid equivalent (GAE)/g (about 3.2-fold)	[42]
papaya	lycopene	solvents: 42.28% ethanol in ethyl acetate conditions: 40 kHz, 800 W, 26.09 min, 50.12 °C	40% ethanol in ethyl acetate (300 mL) 95 °C in a Soxhlet extractor	Recovery of lycopene increased from 68.3 ± 4.1 to 189.8 ± 4.5 μg/g	[43]
carrot	carotenoids	solvents: sunflower oil conditions: 22.5 W/cm^2^, 40 °C, 20 min	hexane at room temperature for one hour	obtained the β-carotene yield of 334.75 mg/L just in 20 min while the CSE method using hexane as solvent obtained the β-carotene yield of 321.36 mg/L after one-hour extraction	[44]
**microwave-assisted extraction (MAE)**					
*Achillea millefolium* dust	antioxidants	solvents: 70% ethanol conditions: 170 W, 40 mL/g, 33 s	40% ethanol at room temperature (1:10, *v*/*v*) for 48 h	increased total polyphenol content from 135.26 ± 1.72 to 237.74 ± 2.08 mg GAE/g, total flavonoid content from 30.82 ± 2.35 to 42.95 ± 1.32 mg quercetin equivalents (QE)/g, 2,2-diphenyl-1-picrylhydrazyl (DPPH) radical scavenging activity from 21.58% ± 0.88% to 71.72% ± 2.12%	[45]
*Quercus* bark	polyphenols	solvents: ethanol content 33%, methanol content 0.38% conditions: 50 Hz, 45 W, 60 min, pH 10.75, room temperature	the same extraction condition without microwave treatment	increased by 3 times and 2 times respectively for total phenolic content and antioxidant recoveries	[46]
**enzyme-assisted extraction (EAE)**					
wine making by-products	phenolics	solvents: 70% acetone enzyme treatment with 2% viscozyme solution stirred for 12 h at 37 °C or 1 mg/mL pronase solution stirred for 1 h, then extraction with 70% (*v*/*v*) acetone in a gyratory water bath shaker at 30 °C for 20 min	the same extraction protocol without enzyme treatment	pronase and viscozyme increased the content of soluble phenolics while reducing the content of insoluble-bound phenolics	[32]
grape skins	flavonoids	solvents: buffer solution containing an appropriate amount of enzyme conditions: 10.52 mg/g Lallzyme EX-V, pH 2.0, extraction at 45 °C for 3 h	70% aqueous ethanol containing 1% formic acid for one day in the dark	improved recovery of anthocyanin contents (from 40,496.19 ± 58.18 to 41,752.95 ± 76.10 mg/kg) and flavan-3-ol contents (from 329.32 ± 2.46 to 345.94 ± 2.88 mg/kg)	[47]
tomato processing waste	lycopene	solvents: hexane/acetone/ethanol (50:25:25 *v*/*v*) conditions: 1.5% cellulase/2% pectinase at 4 h of incubation period	without enzyme treatment	increased the yield of lycopene from less than 200 to 847.33 μg/g (cellulase treatment) and to 1262.56 μg/g (pectinase treatment)	[48]
**pressurized liquid extraction (PLE)**					
Aerial parts of *Dracoceph-alum kotschyi*	phenolics and flavonoids	solvents: methanol conditions: 74 °C, 34 bar pressure, 11.33 min static time, 17.45 min dynamic time, and 0.7 mL/min solvent flow rate	percolated with 1.0 L of methanol at room temperature (25 °C) according to the European Pharmacopeia	improved recovery of total phenolic (from 22.29 ± 0.05 to 30.92 ± 0.03 GAE mg/g), total flavonoids (from 5.042 ± 0.04 to 6.13 ± 0.07 QE mg/g) and luteolin content (from 9550 ± 0.3 to 13,247 ± 0.2 μg/g)	[49]
roots of *Scutellaria pinnatifida*	phenolics and flavonoids	solvents: methanol conditions: 65.8 °C, 39.2 bar pressure, 12.9 min static time, 18.9 min dynamic time, and 0.76 mL/min solvent flow rate	percolated with 1.0 L of methanol at room temperature	the total phenolic content increased from 196.66 to 396.94 mg/g, and the total flavonoid content increased from 91.3 to 127.78 mg/g	[50]
black bamboo leaves	antioxidants	solvents: 50% ethanol for the total phenolic (TP) and 75% ethanol for total flavonoid (TF) and 25% ethanol for DPPH radical scavenging ability conditions: 1500 psi, 200 °C, 25 min static time	reflux extraction method (~90 °C, 1 L solvent, 60 min)	improved extraction yields from 240 to 500 mg/1 g Dry black bamboo leaves (DL), TP contents from 1510 ± 3.2 to 2682 ± 0.9 mg/100 g, TF contents from 182 ± 2.7 to 657 ± 1.7 mg/100 g	[51]
palm pressed fiber	β-carotene	solvents: n-hexane conditions: 80 °C, 1500 psi, 2 × 10 min static extractions with flush volume 50%	extracted with n-hexane and chloroform in a Soxhlet apparatus for 8 h	obtained total β-carotene and vitamin contents comparable to Soxhlet extraction but with lower total organic solvent and rapid extraction process	[52]
**supercritical fluid extraction (SFE)**					
myrtle leaves and berries	antioxidants	solvents: carbon dioxide conditions: 23 MPa, 45 °C and a CO_2_ flow of 0.3 kg/h using absolute ethanol as co-solvent with a flow rate of 0.09 kg/h	obtained by hydrodistillation using a Clevenger-type apparatus, for two hours	increased antioxidant capacity (about 20–40 times), polyphenolic contents (about 2 times) and myricetin-3-*O*-rhamnoside content (about 110–170 times in fruit and about 130–210 times in leaves)	[53]
*Prunus persica* leaves	phenolic compounds	solvents: carbon dioxide conditions: 60 °C, 150 bar and 6% ethanol co-solvent at a flow rate of 15 g/min and for a duration of 60 min	extracted 3 times with 30 mL of solvent system (acetone:methanol:water:formic acid, 40:40:20:0.1)	the radical scavenging activity value increased from 32.23% to 53.25%	[54]
**high hydrostatic pressure extraction (HHPE)**					
prickly pear beverages prepared with 10% peel and 90% pulp	Phyto-chemical Compounds	400 or 550 MPa, room temperature, 0–16 min	thermally treated at 138 ± 1 °C for 2 s	increased TP content (16%–35%) and antioxidant activity (8%–17%) for Cristal (A) and Rojo San Martin varieties as well as increased the betaxanthin contents (6%–8%) and betacyanin content (4%–7%) for Rojo San Martin variety	[55]
*Panax ginseng*	phenolic compounds	600 MPa for 1 min at room temperature	conventional steaming	increased the total phenolic contents (from 1.13 to 1.37 mg maltol equivalent/g of red ginseng), especially maltol content (4.38 to 12.61 mg/100 g of red ginseng), also improved the ferrous ion chelating and superoxide dismutase activities	[56]
**Pulsed electric field extraction (PEFE)**					
defatted canola seed cake	polyphenols	10% ethanol 30 V, 30 Hz and 10 s	microwave processing (5 min, liquid/solid ratio of 6.0 and 633.3 W)	less solvent usage, a shorter extraction time	[57]
*Borago officinalis* L. leaves	polyphenols	acidic water (pH 1.5) 1 to 7 kV/cm, 15–150 μs, 0.04 to 61.1 kJ/kg	the same extraction without PEF treatment	increased the TPC (1.3–6.6 times) and ORAC values (2.0–13.7 times)	[58]
orange peel	polyphenols	distilled water 5 kV/cm, 60 μs, 0.06 to 3.77 kJ/kg, pressurization at 5 bars for 30 min	the same extraction without PEF treatment	increased the naringin content from 1 to 3.1 mg/100 g and hesperidin content from 1.3 to 4.6 mg/100 g	[59]
**high voltage electrical discharges extraction (HVEDE)**					
olive kernel	phenolic compounds	49% ethanol, 66 kJ/kg, pH 2.5	PEF with electric field strength E = 13.3 kV/cm and UAE at 400 W and 24 kHz	more effective polyphenol extraction (255 mg GAE/L for HVEDE versus 140 and 146 mg GAE/L for UAE and PEFE, respectively)	[60]

**Table 2:** Comparison of non-conventional extraction techniques.

Method	Brief Description	Investment Cost	Energy Efficiency	Merits	Drawbacks	Reference
ultrasound-assisted extraction	Sample is extracted with solvent in a vessel and immersed in an ultrasonication bath.	low	medium	fast energy transfer; high extraction yield; low solvent use; short extraction time (5–60 min)	lack of uniformity in the process; generating damages to the ear of the operator; filtration and clean-up step required.	[64,65,144,145]
microwave -assisted extraction	Sample is extracted with a microwave absorbing solvent in a closed/open vessel and irradiated with microwave.	medium	medium	quick heating for bioactive compounds extraction; high extraction yield; low solvent use; moderate extraction time (1 min–40 min)	Extraction solvent must be able to absorb microwaves; filtration and cleanup step required.	[64,75,145]
enzyme-assisted extraction	Sample and enzyme solution are loaded into a vessel and placed in a water bath thermostated at the certain temperature and time.	medium	medium	moderate extraction conditions; eco-friendly; selectivity due to the specificity of enzymes	Expensive cost of enzymes; activity of enzymes varying with the environmental factors; filtration and cleanup step required.	[88,91,94]
pressurized liquid extraction	Sample and solvent are heated and pressurized in a vessel with elevated temperature and pressure. After finishing the extraction, the extract is automatically into a vial.	high	high	high extraction yield; low time and solvent consumption; protection for oxygen and light sensitive compounds; no filtration required; automated systems	clean-up step required; expensive equipment required.	[96,145]
supercritical fluid extraction	Sample is extracted with super-critical fluid in a vessel with high pressure. the analytes are collected in a small volume of solvent or onto a solid-phase trap.	high	high	green solvents (e.g., CO_2_) used; high extraction yield; better separation of solute from solvent; possibility to on-line combining with chromato-graphic process; reduced the thermal degradation; no cleanup or filtration required; automated systems	limited ability to dissolve polar compounds; more parameters to optimize.	[64,106,107,117,145]
high hydrostatic pressure extraction	Sample was mixed with solvent and placed in a sterile poly-ethylene bag, which is eliminated air from the inside and placed into a pressure extractor at different pressure values.	high	high	waste-free process; short time (only about 5 min); performed at room temperature without any heating process	High investment cost and cost-intensive maintenance and service, which make industrial application difficult.	[123,127,146]
pulsed electric field extraction	Extraction was performed between two plate electrodes with 2–3 cm distance and the sample is placed in the treatment chamber.	high	high	mild (non-thermal) processing method; short time (less than 1 s)	Extraction must be applied to food products that can withstand high electric fields and have low electrical conductivity.	[129,146,147]
high voltage electrical discharges extraction	HVED treatment was performed between the stainless steel needle and the grounded plate electrodes with 1 cm distance and the sample is placed in the treatment chamber.	high	high	mild (non-thermal) processing method; high extraction efficiency; short extraction time	High voltage electrical discharges may generate chemical products and free reactive radicals, which can react with antioxidant compounds, thus decreasing their bioactive activity.	[147]

**Table 3:** The antioxidant capacities of some foods and medicinal plants.

Category	Varieties Showing Strong Antioxidant Activities	Assessment Method	Reference
**antioxidant activities at chemical level**			
26 spices	oregano, cinnamon stick, clove, cinnamon, sage	Trolox equivalent antioxidant capacity (TEAC), Folin–Ciocalteu reagent (FCR)	[8]
62 fruits	Chinese date, pomegranate, guava, sweetsop, persimmon, Chinese wampee and plum, grape (red)	TEAC, ferric-reducing antioxidant power (FRAP), FCR	[11]
24 cereal grains	black rice, red rice, purple rice, buckwheat	TEAC, FRAP, FCR	[12]
49 Edible macro-fungi	*Thelephora ganbajun*, *Boletus edulis*, *Volvariella volvacea*, *Boletus regius*, and *Suillus bovinus*	TEAC, FRAP, FCR	[13]
56 vegetables	Chinese toon Bud, loosestrife, perilla leaf, cowpea, caraway, lotus root, sweet potato leaf, soy bean (green), pepper leaf, ginseng leaf, chives, and broccoli	TEAC, FRAP, FCR	[14]
223 medicinal plants	*Acanthopanax gracilistylus*, *Agrimonia pilosa*, *Anemarrhena asphodeloides*, *Caesalpina sappan*, *Carthamus tinctorius*, *Dioscorea bulbifera*, *Fraxinus rhynchophylla*, *Lonicera japonica* (flower), *Magnolia officinalis*, *Mentha haplocalyx*, *Paeonia lactiflora* (red), *Polygonum multiflorum* (Stem), *Polygonum multiflorum* (Root), *Rhodiola sacra*, *Salvia miltiorrhiza*, *Tussilago farfara*, *Sargentodoxa cuneata*	TEAC, FRAP, FCR	[15]
51 edible and wild flowers	*Rosa rugosa*, *Limonium sinuatum*, *Pelargonium hortorum*, *Jatropha integerrima* and *Osmanthus fragrans*, *Orostachys fimbriatu*, *Chaenomeles sinensis*, *Calliandra haematocephala*	TEAC, FRAP, FCR	[16]
50 fruit wastes	grape seed, hawthorn peel, longan peel, longan seed, mango peel, Chinese olive peel and sweetsop peel	TEAC, FRAP, FCR	[19]
**antioxidant activities at cellular level**			
27 vegetables	beet, broccoli, and red pepper, eggplant, Brussels sprout, cabbage	cellular antioxidant activity (CAA) based on HepG2 cells	[183]
25 fruits	pomegranate and berries (wild blueberry, blackberry, raspberry, and blueberry)	CAA based on HepG2 cells	[184]
11 Thai pigmented (red and purple) and 2 nonpigmented rice varieties	hawm dowk mali deang (red), hawm deang sukhothai1 (red), hawm deang (red), man pu (red), red rose (red), klam moang (purple), klam chiang mai (purple)	CAA based on HL-60 cells	[185]
13 food legumes	black soybean, black bean, pinto bean, lentil, green pea, yellow soybean	CAA based on human gastric adenocarcinoma AGS cells	[192]
12 plantago species	*P. lanceolata*, *P. himalaica*, *P. depressa*, *P. cornuti*, *P. jehohlensis*	CAA based on HepG2 cells	[193]
seven cultivars of Aloe	Aloe. *greenii*, Aloe. *arborescens*	CAA based on HepG2 cells	[194]
five main *Phyllanthus emblica* L. cultivars	qingyougan, binggan and boligan	CAA based on HepG2 cells	[195]
